# Relationship between consciousness and the thalamocortical tract in patients with intracerebral hemorrhage

**DOI:** 10.1097/MD.0000000000035510

**Published:** 2023-10-13

**Authors:** Sung Ho Jang, Sung Jun Lee, Min Jye Cho

**Affiliations:** a Department of Physical Medicine and Rehabilitation, College of Medicine, Yeungnam University, Namku, Taegu, Republic of Korea; b Department of Physical Therapy, College of Health Sciences, Dankook University, Dongnamgu, Cheonan, Republic of Korea.

**Keywords:** consciousness, diffusion tensor tractography, Intracerebral hemorrhage, recovery mechanism, thalamocortical tract

## Abstract

In patients with intracerebral hemorrhage (ICH), the relationship between consciousness and the thalamocortical tract (TCT), which links the thalamic intralaminar nuclei (ILN) and the cerebral cortex, was investigated. Forty-one patients with ICH were assigned to 1 of 2 groups according to their preservation of consciousness as determined by their Glasgow coma scale (GCS) score. Patient group A had impaired consciousness (GCS < 15, 21 patients), and patient group B had intact consciousness (GCS = 15, 20 patients). The control group included 20 age- and sex-matched healthy subjects. For all groups, the TCTs from the thalamic ILN of both sides were reconstructed using a probabilistic tractography method based on a multifiber model. In addition, tract volume (TV) values were determined. The TV values for the ipsilateral TCT from the thalamic ILN of the all-patient groups and those for contralateral TCT of the patient group B showed no significant differences between ICH and contra-ICH sides (*P* > .05). The TV results for the ipsilateral and contralateral TCTs from the thalamic ILN of the ICH and contra-ICH sides were significantly different among the 3 groups (*P* < .05). Among the patients, there were moderate positive correlations between GCS scores and TV values of the ipsilateral TCT on the ICH and contra-ICH sides (*R* = 0.477, *P* < .05; *R* = 0.426, *P* < .05). The TV of the ipsilateral TCT from the thalamic ILN on the ICH and contra-ICH sides was significantly correlated with the consciousness level in patients with ICH. Our results could be helpful when developing therapeutic strategies for ICH patients with disorders of consciousness.

## 1. Introduction

Intracerebral hemorrhage (ICH), present in 10% to 15% of stroke patients, is mainly caused by hypertension, cerebral amyloid angiopathy, and vascular malformation.^[1,2]^ Impaired consciousness is a frequent, neurologically severe feature following ICH, and approximately half of patients with ICH are known to experience some form of impaired consciousness.^[2,3]^ Impaired consciousness can cause problems for patients, family members, and society, including a high economic burden due to a loss of working ability and increased medical and caregiving expenses.^[4]^

Consciousness, comprised of arousal and awareness of self and the environment, is not fully understood, but it is reported to be controlled by a complicated series of complex actions that involve a variety of neural structures.^[5,6]^ In addition to the default mode network, fronto-parietal network, fronto-striatal network, and the lower ascending reticular activating system, the thalamocortical tract (TCT) is considered an important neural network involved in consciousness.^[7–12]^ The recently developed diffusion tensor tractography (DTT) approach, which is based on the results of diffusion tensor imaging (DTI), allows the visualization and estimation of neural tracts such as the TCT, which forms a link between the thalamic intralaminar nuclei (ILN) and the cerebral cortex. Detailed visual examination of TCT features and discrimination of the TCT from adjacent neural structures cannot be accomplished when using computed tomography and conventional magnetic resonance imaging.^[13]^

Elucidation of neural correlates to consciousness is clinically important for the management of patients with disorders of consciousness (DOC) because such information can be useful when developing therapeutic strategies for neurorehabilitation in patients with DOC. Previous studies have reported on neural correlates to consciousness in patients with DOC,^[12,14–25]^ and the TCT linking the thalamic ILN and the cerebral cortex has been suggested as an important neural correlate to consciousness in patients with brain injury.^[12,15,19–23,26]^ However, further elucidation of that correlation is needed.

In this study, by applying DTT and assessing the DOC level, we investigated the relationship between consciousness and the TCT from the thalamic ILN in patients with ICH.

## 2. Materials and methods

### 2.1. Subjects

Forty-one patients with ICH (29 men, 12 women; mean age, 47.2 ± 11.9 years; range, 22–68 years) and a randomized group of 20 age- and sex-matched healthy control subjects (9 men, 11 women; mean age, 42.8 ± 11.1 years; range, 27–69 years) with no history of neurologic/psychiatric disease and head trauma were recruited. Among all patients with ICH who visited the department of rehabilitation of a university hospital, 41 patients were recruited according to the following inclusion criteria: First-ever stroke; DTI scans obtained in the early post-ICH period (within 1 month after onset); Age at the time of ICH: 20 to 70 years; Spontaneous ICH was confined to a unilateral supratentorial area and was confirmed by a neuroradiologist, and; No history of neurologic/psychiatric disease and head trauma. The Glasgow coma scale (GCS) was used to determine the consciousness state of the patients at the time of DTI scanning.^[27]^ Based on GCS scores, the patients were divided into 2 groups. Patient group A had impaired consciousness (GCS < 15, 21 patients) and patient group B had intact consciousness (GCS = 15, 20 patients). No significant differences in age-and sex compositions of the patient and control groups were detected (*P* > .05). This retrospective study was performed in accordance with the requirements of the Declaration of Helsinki research guidelines and conducted in accordance with the recommendations of the institutional review board of Yeungnam University Hospital. All of the patients and control subjects signed an informed consent form, and the institutional review board of Yeungnam University Hospital approved the study protocol (ethical approval number: YUMC-2021-03-014).

### 2.2. Diffusion tensor imaging and tractography

**Figure 1.**
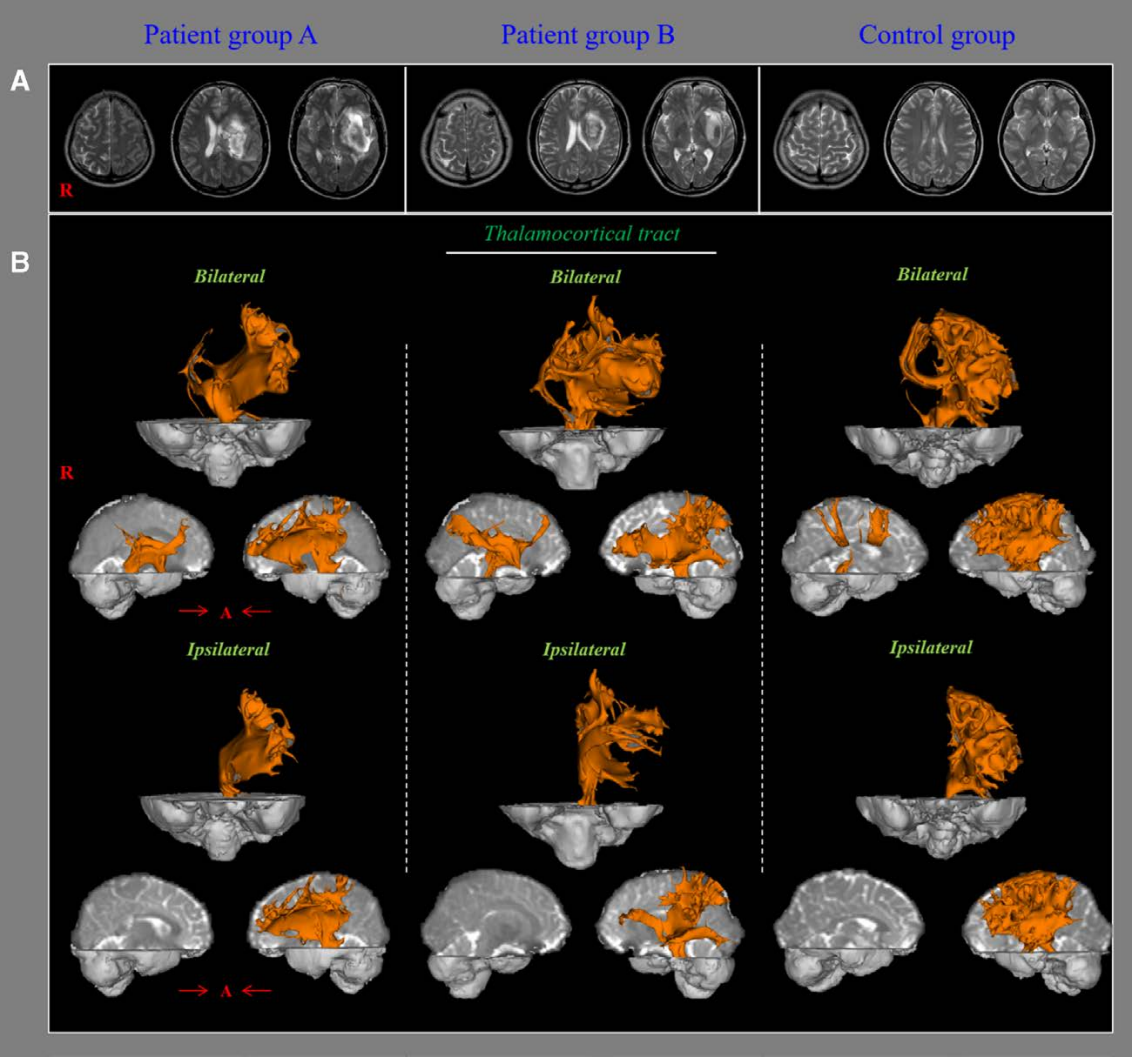
(A) T2-weighted brain magnetic resonance images obtained at the time of diffusion tensor imaging in representative subjects of patient group A (41-year-old male), patient group B (49-year-old male), and the control group (42-year-old male). (B) Results of diffusion tensor tractography of the bilateral and ipsilateral thalamocortical tracts of the left intralaminar nuclei (ILN).

The DTI data were acquired at 14.9 ± 7.1 days after ICH onset using a 6-channel head coil on a 1.5 T Philips Gyroscan Intera scanner (Philips, Ltd, Best, Netherlands) using single-shot echo-planar imaging. Imaging parameters were as follows: acquisition matrix = 96 × 96; reconstructed matrix = 192 × 192; field of view = 240 mm × 240 mm; repetition time = 10,726 ms; echo time = 76 ms; parallel imaging reduction factor (SENSE factor) = 2; EPI factor = 49; b = 1000 seconds/mm^2^; NEX = 1; and slice thickness = 2.5 mm with no gap (acquired isotropic voxel size 1.3 mm × 1.3 mm × 2.5 mm). Diffusion-weighted imaging data were analyzed using analytical tools included in the Oxford Centre for Functional Magnetic Resonance Imaging of the Brain Software Library (www.fmrib.ox.ac.uk/fsl). Affine multi-scale 2-dimensional registration was used for correction of head motion effects and image distortion due to eddy currents. Fiber tracking was performed using a probabilistic tractography method based on a multifiber model and was applied by using the tractography routines implemented in Functional Magnetic Resonance Imaging of the Brain Diffusion software (5000 streamline samples, 0.5 mm step lengths, curvature thresholds = 0.2).^[28]^ For reconstruction of the TCT from the thalamic ILN, a seed region of interest was placed on the thalamic ILN of both the lesional and non-lesional hemispheres at the level of the inter-commissural plane between the anterior and posterior commissures.^[13,29]^ Tract volume (TV) values for the TCT from the thalamic ILN were determined for both the lesional and non-lesional hemispheres. To obtain information on the ipsilateral TCT from the thalamic ILN for each hemisphere, sagittal exclusion in the middle line of the interthalamic adhesion was applied. The TV of the contralateral TCT from each thalamic ILN was determined by subtraction of the TV of the ipsilateral TCT from the TV for the bilateral TCTs of each thalamic ILN (Fig. 1).

### 2.3. Statistical analysis

Data analysis was performed using SPSS software (version 21.0, SPSS Inc., Chicago, IL). The difference of the ipsilateral and contralateral TV values of patients group A and B between ICH and contra-ICH sides was evaluated using the independent *t* test. One-way analysis of variance with Fisher least significant difference post hoc test was used to compare TV values of patient groups A and B and that of the control group. The level of statistical significance was set at *P* < .05. In the case of the control group, because the TV values of the ipsilateral and contralateral TCTs showed no significant differences between each hemisphere (*P* > .05), the all TV values were calculated as the average of bilateral hemisphere (TV values of right hemisphere - ipsilesional: 14815.45 ± 4573.95, contralateral: 4805.80 ± 3269.77; left hemisphere-ipsilesional: 15256.50 ± 4775.83, contralateral: 4304.00 ± 2724.15). The Pearson correlation test was used to determine the level of correlation between GCS score and TCT TV value. As previously described, a correlation coefficient > 0.60 indicated a strong correlation, a correlation coefficient between 0.40 and 0.59 indicated moderate correlation, that between 0.20 and 0.39 indicated a weak correlation, and a correlation coefficient of ≤ 0.19 indicated a very weak correlation.^[30]^

## 3. Results

Results of the comparison of the TV values for the TCTs of patient groups A and B and the control group are summarized in Table 1. The TV values for the ipsilateral TCT from the thalamic ILN of the all-patient groups and those for contralateral TCT of the patient group B showed no significant differences between ICH and contra-ICH sides (*P* > .05), whereas, there was a significant difference in the TV values for the contralateral TCT of the patient group A between ICH and contra-ICH sides (*P* < .05). The TV values of both the ipsilateral and contralateral TCTs from the thalamic ILN on the ICH and contra-ICH sides were significantly different among the 3 groups (*P* < .05). The TV values of the ipsilateral TCTs from the thalamic ILN on both the ICH and contra-ICH sides were significantly lower in-patient group A than in patient group B (*P* < .05). Whereas for the contralateral TCTs, only the TV on the ICH side was significantly lower in-patient group A than in patient group B (*P* < .05).

**Table 1:** Comparison of tract volumes of the thalamocortical tracts of the two patient groups and the control group.

		Ipsilateral tract			Contralateral tract		
		ICH side	Contra-ICH side	*P* value	ICH side	Contra-ICH side	*P* value
TV	Patient A	11581.48 ± 5818.14	10704.57 ± 2946.07	.542	5129.76 ± 3123.87	7711.38 ± 4254.66	.031*
	Patient B	16646.20 ± 4129.19	15758.85 ± 2915.44	.437	8067.60 ± 3884.32	9174.20 ± 4128.60	.388
	Control	15035.98 ± 4534.70			4554.90 ± 2858.70		
	*P* value	.005†,‡	<.001†,‡		<.003†,§	.001‡,§	

Values presented are means ± standard deviations.

ICH = intracerebral hemorrhage, TV = tract volume.

* Statistically significant at *P* < .05.

† Patient A group significantly differs from patient B group per Fisher`s least significant difference post hoc test, *P* < .05.

‡ Patient A group significantly differs from control group per Fisher`s least significant difference post hoc test, *P* < .05.

§ Patient B group significantly differs from control group per Fisher`s least significant difference post hoc test, *P* < .05.

Correlations between GCS scores and the TV values of the ipsilateral TCTs from the thalamic ILN of both the ICH and contra-ICH sides in the patients with ICH are represented in Table 2. The moderate positive correlations was shown between the GCS score and the TV values of the ipsilateral TCTs on both the ICH (*R* = 0.477, *P* < .05) and contra-ICH sides (*R* = 0.426, *P* < .05).^[30]^ Whereas, the GCS score and TV values had no significant correlations for the contralateral TCT from the thalamic ILN on both the ICH and contra-ICH sides (*P* > .05).

**Table 2:** Correlations between GCS score and tract volume of the thalamocortical tracts in patients with ICH.

	Ipsilateral tract		Contralateral tract	
	ICH side	Contra-ICH side	ICH side	Contra-ICH side
GCS	0.477*	0.426*	0.275	0.139

GCS = Glasgow coma scale, ICH = intracerebral hemorrhage.

* Indicates significant correlation between patient GCS score and TV of the thalamocortical tract, *P* < .05. The Pearson correlation test was used to determine the correlation coefficient.

## 4. Discussion

In the current study, by using DTT and assessing DOC, we investigated the relationship between consciousness level and TV of the ipsilateral and contralateral TCTs from the thalamic ILN on the ICH and contra-ICH sides in patients with ICH. Our results can be summarized as follows. First, there was no significant differences in the TV values for the ipsilateral TCT from the thalamic ILN of the all-patient groups and those for the contralateral TCT of the patient group B between the ICH and contra-ICH sides. Second, the TV values for the ipsilateral TCT from the thalamic ILN on both the ICH and contra-ICH sides decreased in the following order: patient group B (intact consciousness), control group, and patient group A (impaired consciousness). Third, the TV values of the contralateral TCT from the thalamic ILN on both sides decreased in the following order: patient group B (intact consciousness), patient group A (impaired consciousness), and the control group. Fourth, the consciousness state of the ICH patients, as reflected by their GCS scores, was moderately correlated with the TV values of the ipsilateral TCT from the thalamic ILN of both the ICH and contra-ICH sides.

Among the various DTT parameters, the TV value indicates the total number of fibers within a neural tract.^[31]^ A decrement in the TV value of a neural tract indicates a decrement in the number of neural fibers in that neural tract and vice versa. In the current study, the results revealing no distinction between the ICH and contra-ICH sides in the TV values for the ipsilateral TCT from the thalamic ILN of the all patients groups and those for the contralateral TCT of the patient group B, and increments in the TV values of the ipsilateral TCT from the thalamic ILN in the both ICH and contra-ICH sides in patient group B (intact consciousness) and of the contralateral TCT from the thalamic ILN on both sides in patient and group A (impaired consciousness) and group B (intact consciousness) compared to the control group appear to indicate that there were ICH-compensatory increments in the neural fibers of each TCT. Furthermore, the moderate correlation between TV value and consciousness state with the ipsilateral TCT from the thalamic ILN of both sides in patient groups appears to suggest the number of neural fibers in the ipsilateral TCT from the thalamic ILN on both the ICH and contra-ICH sides is an important factor associated with the DOC status of patients with ICH.

Several authors have reported that an increase in ipsilateral thalamocortical connectivity contributes to the recovery of impaired consciousness in patients with DOC.^[12,19–23]^ In 2000, Laureys et al^[15]^ demonstrated increased functional connectivity between the thalamic ILN and the ipsilateral prefrontal and anterior cingulate cortices during recovery of consciousness in a vegetative-state patient by using H_2_^15^O positron emission tomography.^[15]^ Since the introduction of DTI, several case studies have demonstrated increased ipsilateral thalamocortical connectivity to various cerebral cortices, including the prefrontal, parietal, anterior cingulate, and basal forebrain, in stroke patients with DOC.^[19–23]^ In 2019, Jang et al^[12]^ demonstrated that consciousness level was strongly correlated with the fractional anisotropy value of the ipsilateral TCT in patients with hypoxic-ischemic brain injury. Recently, Jang et al^[26]^ [2020] reported on a patient with DOC due to infarction in the right intracerebral artery territory; their DTT-based analysis showed increased thalamocortical connectivity from the thalamic ILN on the lesion side to the contra-lesional cortex. The authors suggested that the increased connectivity of the contralateral TCT from the thalamic ILN of the lesion side to the contra-lesional side in this patient might indicate the occurrence of a compensatory phenomenon related to the severe damage of the affected hemisphere.^[26]^ Nevertheless, the present study is the first to demonstrate the contribution of the ipsilateral TCT from the thalamic ILN on both ICH and contra-ICH sides to the control of consciousness in patients with ICH.

However, several limitations of this study should be considered. First, the fiber tracking technique applied during DTT reconstruction is operator dependent.^[32]^ Second, regions of fiber complexity and crossing can prevent full reflection by DTT of the underlying fiber architecture.^[33,34]^ Third, this retrospective study recruited a relatively small number of subjects. Therefore, further prospective studies involving larger numbers of subjects should be undertaken.

In the current study, we observed increases in TV in the ipsilateral TCT from the thalamic ILN on the ICH and contra-ICH sides, and those increases were moderately correlated with the consciousness level in patients with ICH. Our results could be helpful when developing therapeutic strategies for patients with DOC following ICH. Such tract information can be useful in determining an appropriate approach to the application of neuromodulation therapies, such as repetitive transcranial magnetic stimulation, transcranial direct current stimulation, or transcranial alternative current stimulation, which have been used to restore impaired consciousness.^[35–37]^

## Acknowledgments

This work was supported by the National Research Foundation of Korea (NRF) grant funded by the Korean Government(MSIP) (No. 2021R1A2B5B01001386).

## Author contributions

**Conceptualization:** Sung Ho Jang, Sung Jun Lee, Min Jye Cho.

**Data curation:** Sung Ho Jang, Sung Jun Lee, Min Jye Cho.

**Formal analysis:** Min Jye Cho.

**Investigation**: Sung Ho Jang, Sung Jun Lee, Min Jye Cho.

**Methodology:** Sung Ho Jang, Min Jye Cho.

**Supervision:** Sung Ho Jang.

**Visualization:** Sung Jun Lee.

**Writing – original draft:** Sung Jun Lee, Min Jye Cho.

**Writing – review & editing:** Sung Ho Jang.
